# Sports Trauma: From Prevention to Surgery and Return to Sport

**DOI:** 10.3390/healthcare14152347

**Published:** 2026-08-01

**Authors:** Michele Mercurio, Lucrezia Moggio, Umile Giuseppe Longo, Olimpio Galasso

**Affiliations:** 1 Department of Orthopaedic and Trauma Surgery, “Magna Graecia” University of Catanzaro, “Renato Dulbecco” University Hospital, 88100 Catanzaro, Italy; michele.mercurio@unicz.it; 2 Research Center on Musculoskeletal Health, MusculoSkeletalHealth@UMG, “Magna Graecia” University of Catanzaro, 88100 Catanzaro, Italy; 3 Rehabilitation Unit, Ospedale Degli Infermi, ASLBI, 13900 Biella, Italy; 4 Research Unit of Orthopaedic and Trauma Surgery, Department of Medicine and Surgery, Università Campus Bio-Medico di Roma, Via Alvaro del Portillo, 21, 00128 Roma, Italy; 5 Department of Medicine, Surgery and Dentistry, University of Salerno, 84981 Baronissi, Italy

## 1. Introduction

Sports trauma represents an increasingly significant challenge in contemporary sports medicine, affecting athletes across all levels of participation, from recreational to elite sport [1]. The progressive growth in global sports participation, together with the increasing physical and technical demands of modern athletic performance, has led to a higher incidence of acute and overuse musculoskeletal injuries involving the knee, hip, ankle, and shoulder joints [2,3,4]. These injuries are often associated with significant functional impairment, reduced performance capacity, prolonged time away from sport, and, in some cases, long-term joint degeneration [5]. Beyond the physical consequences, sports trauma may also result in psychological distress, fear of reinjury, and reduced confidence during return-to-sport phases, highlighting the need for comprehensive and multidimensional management strategies [6,7].

Traditionally, sports trauma management has been mainly organized around isolated anatomical injuries and their corresponding surgical or conservative treatments. However, contemporary sports medicine increasingly adopts a continuum-based model of care, integrating injury prevention, early diagnosis, surgical or conservative management, rehabilitation, and return-to-sport (RTS) decision-making [8,9]. Within this framework, anatomical localization remains essential but must be interpreted within a broader functional, biomechanical, and sport-specific context.

This Special Issue, “Sports Trauma: From Prevention to Surgery and Return to Sport”, was conceived to provide an integrated overview of current evidence across this continuum. Organizing contributions according to anatomical region and clinical domain, it highlights how advances in prevention strategies, surgical techniques, rehabilitation approaches, and athlete monitoring collectively contribute to improving outcomes in sports-related injuries.

## 2. Recent Developments in the Field

Over the past decade, sports medicine has undergone substantial development due to advances in biomechanics, surgical techniques, rehabilitation science, and digital health technologies. A growing part of the literature has emphasized the multifactorial nature of sports injuries, highlighting the interaction between intrinsic and extrinsic risk factors, neuromuscular control, workload exposure, psychological readiness, and sport-specific demands, while also integrating wearable technologies and data-driven monitoring systems into injury prevention and rehabilitation strategies [10,11,12,13]. Injury prevention strategies have progressively shifted from generalized conditioning programs toward sport-specific and population-specific interventions. Neuromuscular training protocols have demonstrated efficacy in improving movement quality and reducing injury risk, particularly for anterior cruciate ligament (ACL) injuries [6,14,15,16]. At the same time, surgical management has evolved toward minimally invasive and arthroscopic techniques aimed at restoring joint stability and function while minimizing tissue damage and accelerating recovery. Arthroscopic procedures for shoulder instability, hip femoroacetabular impingement, and ankle instability have become increasingly established in clinical practice, reflecting the broader shift toward joint preservation strategies [8,9,17].

Rehabilitation and return-to-sport paradigms have also significantly advanced. Return-to-sport is now increasingly conceptualized as a continuum rather than a single time-based milestone, incorporating objective functional testing, biomechanical evaluation, psychological readiness, and sport-specific performance criteria [18,19,20]. In parallel, digital health technologies, including wearable sensors, tele-rehabilitation platforms, and motion analysis systems, are progressively reshaping athlete monitoring and rehabilitation delivery. These tools offer new opportunities for individualized load monitoring, remote supervision, and data-driven decision-making, although their clinical integration remains heterogeneous [12,21,22].

Despite these advances, important challenges persist, particularly regarding standardization of rehabilitation protocols, heterogeneity in RTS criteria, and the need for more robust long-term outcome data.

## 3. Knowledge Gaps

Despite significant progress across all domains of sports trauma care, several important knowledge gaps remain. A primary limitation concerns the lack of standardized, anatomy-specific frameworks that integrate prevention, treatment, rehabilitation, and return-to-sport decision-making. Although injury mechanisms differ substantially across the knee, hip, ankle, and shoulder, clinical pathways often remain generalized and insufficiently tailored to joint-specific biomechanical demands [8,19]. In knee injuries, particularly ACL and posterior cruciate ligament (PCL) lesions, doubt persists regarding optimal rehabilitation progression and return-to-sport criteria, with substantial variability in clinical practice and a lack of universally accepted, criterion-based decision-making frameworks [23,24,25,26,27]. Despite advances in surgical techniques and conservative management strategies, there is still no consensus on how best to integrate biomechanical, neuromuscular, and psychological factors into individualized RTS decision-making models. Hip-related injuries, such as femoroacetabular impingement, continue to raise questions regarding long-term joint preservation, optimal timing for intervention, and predictors of return to pre-injury performance [28,29,30]. Similarly, in shoulder instability with significant bone loss, further evidence is needed to better define long-term outcomes and sport-specific functional thresholds following surgical stabilization [31]. At the ankle, although arthroscopic techniques have expanded therapeutic options, high-quality comparative studies and long-term follow-up data remain limited, particularly regarding reinjury prevention and functional durability [32].

Across all anatomical regions, rehabilitation and return-to-sport represent the most variable phases of care, with persistent reliance on heterogeneous and often time-based criteria rather than multidimensional, individualized assessment frameworks [33,34,35,36]. This contributes to ongoing variability in reinjury rates, particularly in high-demand athletic populations. Psychological factors, including fear of reinjury, confidence, and motivational readiness, remain insufficiently integrated into clinical decision-making despite their recognized impact on recovery and performance outcomes [37,38,39]. Finally, although digital health technologies and wearable systems are increasingly used in sports medicine, their standardization, validation, and integration into clinical pathways remain incomplete.

Addressing these gaps will require more integrated, multidisciplinary, and anatomy-informed approaches capable of combining physical, psychological, and technological dimensions within a unified model of sports trauma care.

## 4. Contributions in This Special Issue

The studies included in this Special Issue can be organized according to anatomical region and clinical domain, reflecting the continuum from prevention to surgical management, rehabilitation, and return-to-sport strategies.

### 4.1. Knee Joint: Prevention, Surgical Management, and Rehabilitation

The knee joint was one of the most frequently addressed anatomical regions. From a preventive perspective, Contribution 6 investigated the FIFA 11+ training program, demonstrating its effectiveness in improving neuromuscular control and reducing knee valgus loading in academy soccer players across a competitive season, reinforcing the role of structured injury prevention strategies in reducing ACL injury risk.

In ACL reconstruction, Contribution 5 evaluated outcomes following the modified transtibial technique, reporting favorable functional outcomes and return-to-sport potential in athletes. Complementarily, Contribution 3 examined biomechanical, anthropometric, and temporal factors associated with return to sport after ACL reconstruction, highlighting the multifactorial nature of RTS decision-making and the need for individualized assessment beyond time-based criteria.

Conservative and early-phase management of ligamentous injuries was addressed in Contribution 1, which explored the effectiveness of a dynamic brace in acute PCL lesions. The findings suggested that dynamic bracing may reduce posterior tibial translation while minimizing complications associated with rigid immobilization, supporting its role in early functional preservation strategies.

### 4.2. Hip Joint: Arthroscopy and Functional Outcomes

The systematic review and meta-analysis presented in Contribution 8 evaluated outcomes of hip arthroscopy for femoroacetabular impingement (FAI) in athletes versus non-athletes. The study demonstrated that arthroscopic management provides significant functional improvement and high rates of return to sport, supporting its role in joint preservation and early functional recovery in active populations.

### 4.3. Shoulder Joint: Instability Management

Contribution 4 reported a case series on the congruent-arc Latarjet procedure using a subscapularis split approach for anterior shoulder instability with significant bone loss. The findings highlighted encouraging outcomes in restoring shoulder stability and enabling return to sport in high-demand athletes, supporting the evolution of advanced surgical techniques in shoulder instability management.

### 4.4. Ankle Joint: Arthroscopic Treatment

The systematic review presented in Contribution 9 focused on arthroscopic management of medial or rotational ankle instability. The study emphasized the growing role of minimally invasive techniques as alternatives to open procedures, aiming to improve joint stability while facilitating rehabilitation and return to activity.

### 4.5. Rehabilitation and Return-to-Sport Innovation

Contribution 2 investigated the effects of a multi-faceted training program in senior pickleball players who had previously experienced falls during play or reported self-limiting participation due to fear of falling (FOF). The 10-week intervention combined strength, balance, change-of-direction, and agility training and resulted in significant improvements in change-of-direction performance and reductions in FOF. Participants in the intervention group also reported greater participation in pickleball and a lower incidence of falls during follow-up compared with controls. These findings highlight the value of targeted exercise-based interventions for injury prevention and functional performance enhancement in older recreational athletes, emphasizing the importance of addressing both physical and psychological factors to support safe sport participation.

Contribution 10 reported a case study on telerehabilitation following meniscus surgery in a triathlete. The study highlighted the potential of digital rehabilitation approaches, including remote monitoring and telemedicine tools, to support individualized recovery pathways and facilitate return to sport when traditional rehabilitation may be limited.

### 4.6. Cardiovascular Evaluation and Athlete Safety

Finally, Contribution 7 provided a comprehensive review of Italian COCIS protocols for cardiological evaluation in competitive football players. This contribution expands the scope of sports trauma beyond the musculoskeletal system, emphasizing the importance of cardiovascular screening and pre-participation assessment in ensuring athlete safety.

Collectively, these contributions reflect the progressive integration of preventive strategies, surgical innovation, rehabilitation approaches, and comprehensive athlete monitoring within a unified and multidisciplinary framework of sports trauma care.

## 5. Future Research Directions

Future research should focus on the development of integrated, anatomy-specific models capable of combining injury prevention, surgical decision-making, rehabilitation, and return-to-sport assessment. Priority should be given to improving the objectivity and individualization of RTS criteria through the integration of biomechanical analysis, neuromuscular testing, psychological readiness, and sport-specific performance indicators. Longitudinal studies are also needed to better understand long-term outcomes following both conservative and surgical interventions, including reinjury rates, joint degeneration, and functional performance over time. Digital health technologies, including wearable sensors, tele-rehabilitation systems, and artificial intelligence-based monitoring tools, represent promising areas for future development but require stronger validation and standardization before widespread clinical adoption. Greater attention should also be given to psychological factors and their integration into rehabilitation pathways, particularly regarding fear of reinjury and confidence restoration. Finally, future studies should better address underrepresented populations, including recreational athletes, female athletes, and older individuals, to ensure more inclusive and generalizable evidence.

## 6. Conclusions

Sports trauma management has evolved from isolated injury treatment toward a comprehensive, multidisciplinary continuum encompassing prevention, diagnosis, surgical and conservative management, rehabilitation, and return-to-sport decision-making.

The studies included in this Special Issue collectively illustrate this transition, highlighting advances in injury prevention strategies, minimally invasive surgical techniques, individualized rehabilitation approaches, tele-rehabilitation, and comprehensive athlete monitoring. Despite these advances, important challenges remain regarding the standardization of clinical pathways, integration of psychological assessment, and the implementation of digital health technologies into routine practice. Future progress will depend on the development of more personalized, multidisciplinary, and technology-assisted approaches aimed not only at restoring anatomical integrity but also at optimizing long-term athlete health, performance, and safe return to sport.
